# Consistency of tumor and immune cell programmed cell death ligand-1 expression within and between tumor blocks using the VENTANA SP263 assay

**DOI:** 10.1186/s13000-018-0725-9

**Published:** 2018-07-24

**Authors:** Paul Scorer, Marietta Scott, Nicola Lawson, Marianne J. Ratcliffe, Craig Barker, Marlon C. Rebelatto, Jill Walker

**Affiliations:** 1 Precision Medicine Laboratories, Precision Medicine and Genomics, IMED Biotech Unit, AstraZeneca, HODGKIN, C/O B310 Cambridge Science Park, Milton Road, Cambridge, CB4 0WG UK; 2 Oncology Companion Diagnostics Unit, Precision Medicine and Genomics, IMED Biotech Unit, AstraZeneca, Cambridge, UK; 3 Translational Sciences, Research, MedImmune, Gaithersburg, MD USA

**Keywords:** PD-L1, Heterogeneity, Assay, Concordance, Consistency, Reproducibility, Immunohistochemistry, SP263, Intra-block, Intra-case

## Abstract

**Background:**

Several anti-programmed cell death-1 (PD-1) and anti-programmed cell death ligand-1 (PD-L1) therapies have shown encouraging safety and clinical activity in a variety of tumor types. A potential role for PD-L1 testing in identifying patients that are more likely to respond to treatment is emerging. PD-L1 expression in clinical practice is determined by testing one tumor section per patient. Therefore, it is critical to understand the impact of tissue sampling variability on patients’ PD-L1 classification.

**Methods:**

Resected non-small cell lung cancer (NSCLC), head and neck squamous cell carcinoma (HNSCC) and urothelial carcinoma (UC) tissue samples (five samples per tumor type) were obtained from commercial sources and two tumor blocks were taken from each. Three sections from each block (~ 100 μm apart) were stained using the VENTANA PD-L1 (SP263) assay, and scored based on the percentage of PD-L1-staining tumor cells (TCs) or tumor-infiltrating immune cells (ICs) present. Each section was categorized as PD-L1 high or low/negative using a variety of cut-off values, and intra-block and intra-case (between blocks of the same tumor) concordance (overall percentage agreement [OPA]) were evaluated. An additional 200 commercial NSCLC samples were also analyzed, and intra-block concordance determined by scoring two sections per sample (≥70 μm apart).

**Results:**

Concordance in TC PD-L1 classification was high at all applied cut-offs. Intra-block and intra-case OPA for the 15 NSCLC, HNSCC or UC samples were 100% and 80–100%, respectively, across all cut-offs; intra-block OPA for the 200 NSCLC samples was 91.0–98.5% across all cut-offs. IC PD-L1 classification was less consistent; intra-block and intra-case OPA for the 15 NSCLC, HNSCC or UC samples ranged between 70 and 100% and between 60 and 100%, respectively, with similar observations in the intra-block analysis of the 200 NSCLC samples.

**Conclusions:**

These results show the reproducibility of TC PD-L1 classification across the depth of the tumor using the VENTANA PD-L1 (SP263) assay. Practically, this means that treatment decisions based on TC PD-L1 classification can be made confidently, following analysis of one tumor section. Although more variable than TC staining, consistent IC PD-L1 classification was also observed within and between blocks and across cut-offs.

**Electronic supplementary material:**

The online version of this article (10.1186/s13000-018-0725-9) contains supplementary material, which is available to authorized users.

## Background

Many tumors evade detection by the immune system by exploiting inhibitory pathways (checkpoints) that suppress antitumor responses [1]. Antibodies have been developed that target these checkpoints with the aim of restoring antitumor immune activity. One of the most promising targets is the programmed cell death-1 (PD-1) / programmed cell death ligand-1 (PD-L1) checkpoint pathway, which negatively regulates effector T-cell activity, inhibiting antitumor immune responses and thereby promoting tumor immune evasion [2, 3].

The anti-PD-1 therapies pembrolizumab and nivolumab and the anti-PD-L1 agents durvalumab, atezolizumab and avelumab have demonstrated antitumor activity and manageable safety profiles across different tumor types [4–15]. Evidence suggests that these types of therapies are associated with higher response rates in patients whose tumors have high PD-L1 expression compared to those with low/no PD-L1 expression [4, 5, 10, 16–18]. Some of these agents are now available with companion or complementary PD-L1 diagnostic assays in various indications [19–22]; use of these assays aims to inform treatment decisions by identifying patients who are most likely to respond to treatment.

The clinical assessment of PD-L1 status relies on testing one formalin-fixed paraffin-embedded (FFPE) section per patient. Selection of a tumor section for biomarker analysis, including testing for PD-L1, may be random or dependent on factors such as sample quality or tumor tissue availability. Variations in the populations of PD-L1-staining tumor cells (TCs) and/or tumor-infiltrating immune cells (ICs) within a tumor could potentially impact the classification of the tumor as PD-L1-high or PD-L1-low/negative.

Cellular architecture and IC infiltration can vary throughout the tumor; however, the impact of this on PD-L1 expression levels and, more importantly, the PD-L1 status used in assessing patient suitability for certain treatments, is not fully understood. A study by Rehman et al. investigating the heterogeneity of PD-L1 expression in non-small cell lung cancer (NSCLC) tumor samples showed variability in PD-L1 expression between fields of view on the same slide (91% variance for TCs), but minimal heterogeneity between different blocks of the same tumor (94% concordance for TCs) [23]. However, while the Rehman et al. study provides information about intra-section and intra-case heterogeneity, the variability within a single tissue block (intra-block) was not investigated.

Data on intra-block and intra-case concordance in PD-L1 classification are available for the VENTANA PD-L1 (SP142) assay, and the Dako PD-L1 IHC 28–8 PharmDx and PD-L1 IHC 22C3 PharmDx assays, in NSCLC and urothelial carcinoma (UC) tissue samples [24–27]. The objective of our study was to assess the intra-block and intra-case concordance in PD-L1 staining of TC and IC populations using the VENTANA PD-L1 (SP263) assay. Tissue samples from NSCLC, head and neck squamous cell carcinoma (HNSCC) and UC were assessed.

## Methods

### Tumor samples, preparation and staining, and assessment of 15 NSCLC, HNSCC or UC samples

For this study, FFPE samples of resected tissue from primary NSCLC, HNSCC and UC tumors were obtained from commercial sources (Avaden Biosciences, Seattle WA, USA). Appropriate patient consents for sample use were in place. To ensure the sample cohort covered a wide range of PD-L1 TC expression, FFPE sections were acquired for 20 cases from each indication (60 cases in total) and stained with the VENTANA PD-L1 (SP263) assay. Five representative cases were then selected from each indication (15 cases in total). The 15 selected cases included six cases with PD-L1 expression in > 70% of TCs, six cases with PD-L1 expression in 20–50% of TCs, and three cases with little or no PD-L1 expression in TCs. This selection was performed independently, prior to circulation of the slides to the study pathologist. The 15 cases were selected primarily on TC content and PD-L1 expression in TCs; the IC content and PD-L1 expression in ICs were assessed for confirmation that ICs would be present for analysis. From the 15 cases entered into the study, 14 had PD-L1 expression in < 10% of ICs and one had PD-L1 expression in > 20% of ICs.

**Fig. 1:**
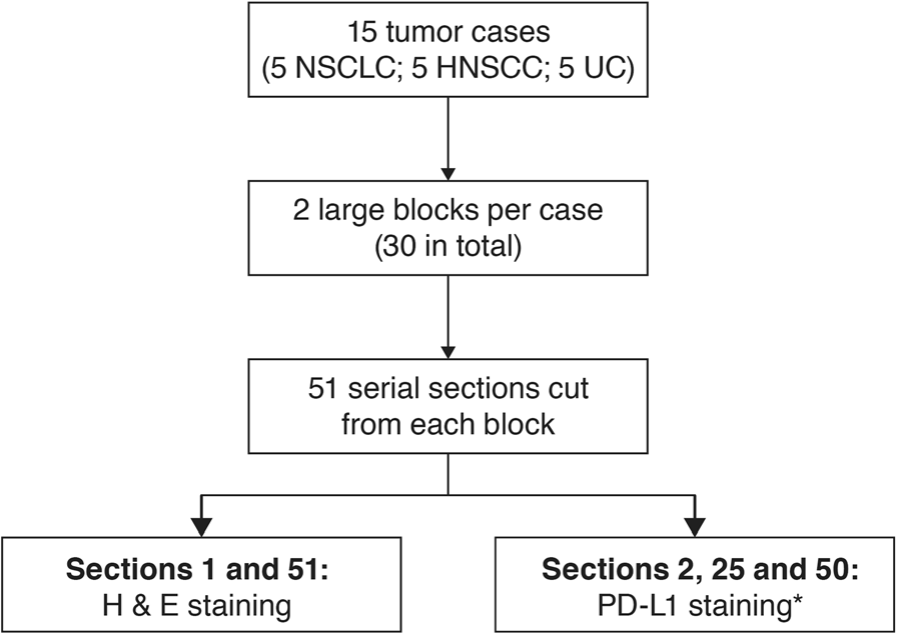
Sample preparation from 15 NSCLC, HNSCC or UC cases. *Using the VENTANA PD-L1 (SP263) assay with the VENTANA OptiView DAB IHC detection kit on the automated VENTANA BenchMark ULTRA platform. H & E: hematoxylin and eosin; HNSCC: head and neck squamous cell carcinoma; NSCLC: non-small cell lung cancer; PD-L1: programmed cell death ligand-1; UC: urothelial carcinoma

Following the initial screening, two large tumor resection blocks were taken from each case (30 blocks in total). The samples were sectioned serially at 4 μm on to Superfrost Plus slides, dried at room temperature or 37 °C overnight and then baked at 56 °C for 1 h. Fifty-one serial sections were cut fresh from each block (Fig. 1) and cut sections were stored in slide storage boxes with close-fitting lids at − 20 °C until stained (within 1 month). Sections “Background” and 51 from each block were stained with hematoxylin and eosin to confirm that tumor was present in all serial sections, and to ensure there were enough TCs in the sections to provide an accurate estimate of PD-L1 expression. Sections “Methods”, 25 and 50 from each block were stained using the VENTANA PD-L1 (SP263) assay with the VENTANA OptiView DAB IHC Detection Kit on the automated VENTANA BenchMark ULTRA platform. The sections were ~ 100 μm apart.

**Fig. 2:**
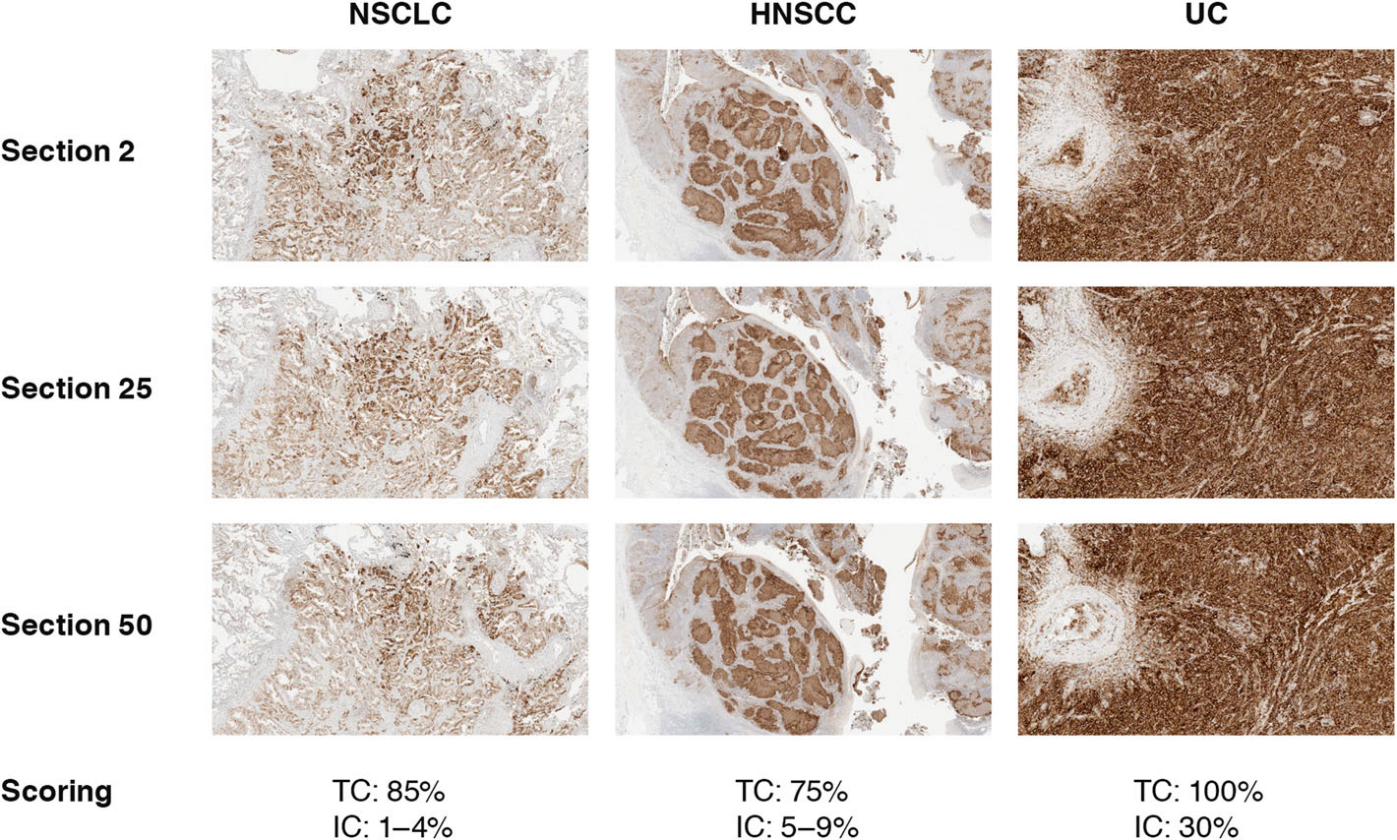
PD-L1 antibody staining in tumor tissue samples. IHC images of PD-L1 staining (using the VENTANA PD-L1 [SP263] assay with the VENTANA OptiView DAB IHC detection kit on the automated VENTANA BenchMark ULTRA platform) in NSCLC, HNSCC and UC tissue samples (three sections from one block of each). The score given to the PD-L1 TC and IC staining by the pathologist is given under the images. Magnification: NSCLC: ×4; HNSCC: ×2; UC: ×10. HNSCC: head and neck squamous cell carcinoma; IC: tumor-infiltrating immune cells; IHC: immunohistochemistry; NSCLC: non-small cell lung cancer; PD-L1: programmed cell death ligand-1; TC: tumor cells; UC: urothelial carcinoma

Stained sections were assessed by a single, certified pathologist, trained in PD-L1 immunohistochemistry interpretation (TCs and ICs) by VENTANA. To minimize bias, the pathologist was blind with respect to the case, block and section number being scored. The total percentage of TCs or ICs that stained positive for PD-L1 was recorded*.* Scoring of TC membrane positivity or IC positivity was performed as per the SP263 scoring algorithm and interpretation guide [28]. Scores of > 10% were recorded in 5% increments; scores of ≤10% were recorded as < 1%; 1–4%, 5–9 and 10% (Fig. 2).

### Staining and assessment of additional 200 NSCLC samples

In addition to the 15 NSCLC, HNSCC or UC cases described above, 200 commercial NSCLC patient samples from Stage I–IV primary tumors (Asterand, MI, USA; ProteoGenex, CA, USA; Tissue Solutions, CA, USA) were available as part of a larger comparative study [29]. The methodology for preparation and assessment of these samples was published previously [29]. Fresh sections were cut from each block, 7 months apart, to simulate repeat testing in a clinical setting. The sections were cut at a minimum of 70 μm separation, and were stained using the VENTANA PD-L1 (SP263) assay. These samples were read by a single blinded pathologist trained by VENTANA in a Clinical Laboratory Improvement Amendments (CLIA) program certified laboratory (Hematogenix, IL, USA). The mean washout period between assessments of the two sections from the same sample was 258 days (range 242–287). PD-L1 positivity was scored as follows: for TCs, scores of > 25% were recorded in 10% increments and scores of ≤25% were recorded as < 1%; 1–4%, 5–9, 10, 20% or 25%; for ICs, scores of > 10% were recorded in 10% increments and scores of ≤10% were recorded as 0, 1, 5% or 10%.

### Statistical analysis: Intra-block and intra-case assessment of 15 NSCLC, HNSCC or UC samples

For the 15 NSCLC, HNSCC or UC cases, TC and IC PD-L1 expression scores from the three sections of each block were recorded (90 TC and 90 IC scores in total; Additional file 1). Multiple clinically relevant diagnostic cut-offs (Table 1) [4, 8, 10–13, 17, 19–22, 30–33] were then applied to the TC and IC scores, and each section was classified as being above or below each cut-off value (PD-L1 high or low/negative status, respectively). The applied cut-offs were: ≥50%, ≥25%, ≥10% and ≥ 1% for TCs; ≥25%, ≥10%, ≥5% and ≥ 1% for ICs.

**Table 1:** Comparison of approved PD-L1 diagnostic assays and PD-L1 cut-offs in NSCLC, HNSCC and UC

	VENTANA SP263 [19, 30]	Dako 22C3 [21]	Dako 28–8 [20]	VENTANA SP142 [22]
Developed as companion diagnostic assay for:	Durvalumab (AstraZeneca/ MedImmune)^a^	Pembrolizumab (Merck Sharp & Dohme)	Nivolumab (Bristol-Myers Squibb)	Atezolizumab (Genentech/Roche)
Compartment	TC; TC or IC	TC; TC & IC	TC	TC or IC; IC
PD-L1 cut-off NSCLC	≥25% TC [30]	≥50% TC - 1 L [21, 31] ≥1% TC - 2 L [31]	≥1%, ≥5%, ≥10% TC [8]	≥50% TC or ≥ 10% IC [13, 22]
PD-L1 cut-off HNSCC	≥25% TC [30]	≥1, ≥50 CPS^b^ [17]	≥1%, ≥5%, ≥10% TC [10]	–
PD-L1 cut-off UC	≥25% TC or IC [4]	≥10 CPS^b^ [32]	≥1%, ≥5% TC [11]	≥5% IC [12, 22]
FDA regulatory status	Approved complementary diagnostic in UC	Approved companion diagnostic in NSCLC	Approved complementary diagnostic in NSCLC	Approved complementary diagnostic in NSCLC and UC

^a^VENTANA SP263 is also approved for use with nivolumab and pembrolizumab in NSCLC patients (CE mark only; not FDA approved) [19]

^b^Previously reported as, and equivalent to ≥1%, ≥10% or ≥ 50% CPS [33]

*CPS* combined positive score evaluating both TC and IC, *HNSCC* head and neck squamous cell carcinoma, *IC* tumor-infiltrating immune cell, *NSCLC* non-small cell lung cancer, *PD-L1* programmed cell death ligand-1, *TC* tumor cell, *UC* urothelial carcinoma

A block was classified as discordant if there was any variation in the diagnostic results (PD-L1 status) for any of its three sections. Intra-case comparisons were the same as intra-block comparisons, with the exception that six sections in total were compared per case (three sections per block; two blocks per case). Overall percentage agreement (OPA) within blocks (intra-block) and between blocks (intra-case) was calculated at each cut-off.

### Statistical analysis: Intra-block analysis of 200 NSCLC cases

The analysis plan for the 200 NSCLC cases was published previously [29]. OPA, negative percentage agreement (NPA) and positive percentage agreement (PPA) [34] were calculated at multiple clinically relevant cut-offs (≥50%, ≥25%, ≥10% and ≥ 1%) for the two sections from each block. For each metric, the lower boundary of the 95% confidence interval (CI) was calculated with no upper bound, using the Clopper-Pearson method [35].

## Results

### Sample demographics

The patient demographics for the 15 NSCLC, HNSCC or UC cases are presented in Table 2. Patient age at the time of surgery ranged between 49 and 82 years. The tumor samples analyzed were at different stages of disease; NSCLC: Stage IB–IIIA; HNSCC: Stage I–III; UC: Stage II–IV. Sample age at the time of analysis ranged from 1 to 13 years. The patient demographics for the 200 NSCLC samples have been presented previously [36]. Thirty-eight percent were Stage I, 36% were Stage II and 21% were Stage III.

**Table 2:** Demographics of patients who provided samples for analysis

Patient	Sample type	Sample age, years	Patient age at surgery, years	Sex	Primary diagnosis	Clinical stage
*NSCLC*						
1	Lung	9	79	Male	Squamous cell carcinoma	II
2	Lung	2	50	Male	Squamous cell carcinoma	IIIA
3	Lung	1	82	Male	Adenocarcinoma	IIB
4	Lung	13	70	Female	Adenocarcinoma	IB
5	Lung	13	71	Male	Squamous cell carcinoma	IIA
*HNSCC*						
6	Tonsil	10	64	Female	Squamous cell carcinoma	I
7	Tongue	9	71	Female	Squamous cell carcinoma	I
8	Larynx	6	55	Male	Squamous cell carcinoma	III
9	Tonsil	6	49	Female	Squamous cell carcinoma	I
10	Tongue	3	68	Male	Squamous cell carcinoma	I
*UC*						
11	Bladder	7	74	Female	Urothelial cell carcinoma	III
12	Bladder	4	70	Male	Urothelial cell carcinoma	II
13	Bladder	4	67	Male	Urothelial cell carcinoma	IV
14	Bladder	3	80	Female	Urothelial cell carcinoma	III
15	Bladder	3	82	Male	Urothelial cell carcinoma	III

*HNSCC* head and neck squamous cell carcinoma, *NSCLC* non-small cell lung cancer, *UC* urothelial carcinoma

### Intra-block concordance in PD-L1 classification

In the analysis of TCs, PD-L1 classification (above or below the cut-off) was consistent within tissue blocks for all the applied cut-offs (TC intra-block OPA was 100%) (Table 3).

**Table 3:** Intra-block concordance (OPA) in PD-L1 classification at various applied cut-offs

Applied cut-off	Concordance (OPA), %		
	NSCLC	HNSCC	UC
*TC PD-L1 staining*			
≥ 50%	100	100	100
≥ 25%	100	100	100
≥ 10%	100	100	100
≥ 1%	100	100	100
*IC PD-L1 staining*			
≥ 25%	100	100	100
≥ 10%	70	90	100
≥ 5%	90	100	80
≥ 1%	100	100	80

Fifteen NSCLC, HNSCC or UC cases; 30 blocks in total (10 blocks per indication)

*HNSCC* head and neck squamous cell carcinoma, *IC* tumor-infiltrating immune cell, *NSCLC* non-small cell lung cancer, *OPA* overall percentage agreement, *PD-L1* programmed cell death ligand-1, *TC* tumor cell, *UC* urothelial carcinoma

PD-L1 classification was less consistent in the analysis of ICs. IC intra-block OPA ranged between 70 and 100% across all tumor types at the ≥1%, ≥5% and ≥ 10% cut-offs. However, OPA was 100% across all tumor types at the ≥25% cut-off (Table 3), reflective of the fact that the majority (14/15) of cases had IC staining scored below 25% (Additional file 1). The percentage of PD-L1-staining ICs between sections of the discordant blocks varied by no more than one scoring category (~ 5%), and the differences in PD-L1 scoring were either: < 1% vs 1–4%; 1–4% vs 5–9% or 5–9% vs 10% (Additional file 1).

**Fig. 3:**
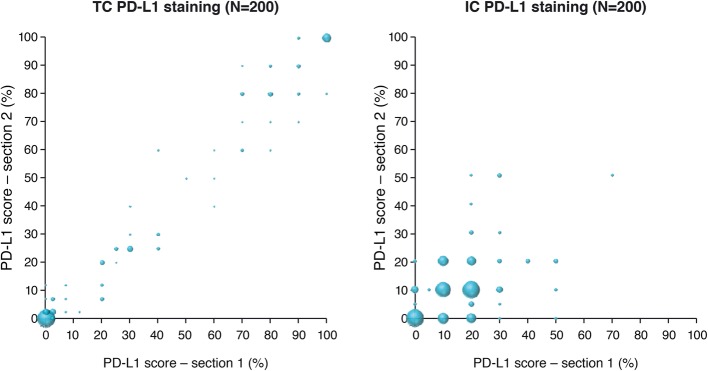
Correlation in PD-L1 staining between two sections from the same tumor block. Sample size of 200 NSCLC cases. Using the VENTANA PD-L1 (SP263) assay with the VENTANA OptiView DAB IHC detection kit on the automated VENTANA BenchMark ULTRA platform. IC: tumor-infiltrating immune cells; NSCLC: non-small cell lung cancer; PD-L1: programmed cell death ligand-1; TC: tumor cells

These results were supported by the analysis of 200 additional NSCLC cases. In this much larger cohort, the minimum intra-block TC OPA was 91.0% (at the ≥1% cut-off); TC PPA and NPA were > 90 and > 80%, respectively, at all cut-offs (range: 81.4–100.0% across both PPA and NPA) (Table 4 and Fig. 3). The highest agreement was observed at the ≥25% cut-off (OPA: 98.5%; PPA: 96.7%; NPA: 100.0%).

**Table 4:** Intra-block concordance (OPA; PPA; NPA) in PD-L1 classification of NSCLC samples at various applied cut-offs

Applied cut-off	OPA % (lower 95% CI)	PPA % (lower 95% CI)	NPA % (lower 95% CI)
*TC PD-L1 staining*			
≥ 50%	97.0 (94.2)	92.2 (84.3)	99.3 (96.6)
≥ 25%	98.5 (96.2)	96.7 (91.6)	100.0 (97.3)
≥ 10%	96.5 (93.5)	95.3 (90.4)	97.8 (93.4)
≥ 1%	91.0 (86.9)	96.2 (92.1)	81.4 (72.1)
*IC PD-L1 staining*			
≥ 50%	95.5 (92.3)	14.3 (0.7)	98.4 (96.0)
≥ 25%	86.5 (81.9)	17.9 (7.3)	97.7 (94.8)
≥ 10%	78.5 (73.2)	78.1 (71.9)	79.6 (67.8)
≥ 1%	80.5 (75.3)	81.6 (75.6)	77.1 (64.9)

200 NSCLC cases (two sections were scored for each case)

*CI* confidence interval, *IC* tumor-infiltrating immune cell, *NPA* negative percentage agreement, *NSCLC* non-small cell lung cancer, *OPA* overall percentage agreement, *PD-L1* programmed cell death ligand-1, *PPA* positive percentage agreement, *TC* tumor cell

PD-L1 classification in ICs was less consistent across this larger sample set as well, with OPA values ranging from 78.5 to 95.5%, across the different cut-offs (Table 4 and Fig. 3). This is also reflected in the PPA and NPA values; PPA values ranged from 14.3% (at the ≥50% cut-off) to 81.6% (at the ≥1% cut-off) and NPA values ranged from 77.1% (at the ≥1% cut-off) to 98.4% (at the ≥50% cut-off) (Table 4 and Fig. 3).

### Intra-case concordance in PD-L1 classification

There was high agreement in TC PD-L1 classification between two different blocks from the same tumor; TC intra-case OPA was 100% across all tumor types and at all applied cut-offs except for NSCLC at the ≥50% cut-off, where OPA was 80% (Table 5).

**Table 5:** Intra-case concordance (OPA) in PD-L1 classification at various applied cut-offs

Applied cut-off	Concordance (OPA), %		
	NSCLC	HNSCC	UC
*TC PD-L1 staining*			
≥50%	80	100	100
≥25%	100	100	100
≥10%	100	100	100
≥1%	100	100	100
*IC PD-L1 staining*			
≥25%	100	100	100
≥10%	60	80	100
≥5%	80	100	80
≥1%	100	80	60

Fifteen NSCLC, HNSCC or UC cases (five cases per indication)

*HNSCC* head and neck squamous cell carcinoma, *IC* tumor-infiltrating immune cell, *NSCLC* non-small cell lung cancer, *OPA* overall percentage agreement, *PD-L1* programmed cell death ligand-1, *TC* tumor cell, *UC* urothelial carcinoma

Intra-case PD-L1 classification in the analysis of ICs was less consistent, with OPA values ranging from 60 to 100% across all tumor types at the ≥1%, ≥5% and ≥ 10% cut-offs (Table 5). However, as with the intra-block analysis, intra-case OPA was 100% across all tumor types at the ≥25% cut-off, again reflecting the lower levels of PD-L1 expression in ICs, compared with TCs.

## Discussion

Clinical data suggest that anti-PD-1 / anti-PD-L1 treatment may be more effective in patients whose tumors have high expression of PD-L1 vs those with low/no expression of PD-L1 [4, 5, 10, 16–18]; as such, it is critical to understand the impact of tissue sampling variability on patients’ PD-L1 classification. Our study analyzed PD-L1 expression in 15 tumor samples from three indications (NSCLC, HNSCC or UC) as well as in a large, separate cohort of 200 NSCLC samples, and is the first study of PD-L1 heterogeneity using the VENTANA SP263 assay. In the analysis of TCs, we showed high intra-block and intra-case concordance in PD-L1 classification (above or below the cut-off value) across all applied cut-offs and for both sets of samples. Our findings are consistent with previously published data [24, 25], and give a high level of confidence in the reproducibility of TC scoring across the depth of the tumor.

The results from the analysis of PD-L1 expression in ICs were not as consistent as those for TCs, with a good to moderate intra-block and intra-case agreement across the applied cut-offs for the 15 NSCLC, HNSCC or UC samples. Despite this increased variability, the intra-block and intra-case OPA for ICs were 100% at the ≥25% cut-off. Whilst only one sample (a UC case) was scored above 25% for ICs, the 100% OPA reflects the fact that there were no large differences in IC scoring within or between blocks for any of the three indications. The ≥25% cut-off is the approved value for the IC component of the scoring algorithm used with the VENTANA PD-L1 (SP263) assay for identifying UC patients most likely to respond to durvalumab [4, 28] and the reproducibility in this small dataset supports the use of this cut-off. In line with these data, intra-block PD-L1 expression was also more variable in ICs than in TCs in the larger NSCLC sample set. The PPA values reported varied from 14.3 to 81.6%; however, the two lowest PPA values at the ≥50% (14.3%) and ≥ 25% (17.9%) cut-offs could be driven by the fact that very few cases were scored above these two cut-off values. The increased intra-case variability in PD-L1 expression in ICs is consistent with a recent study in NSCLC by Rehman et al., who also speculated that the low numbers of PD-L1-expressing ICs may have affected their results [23]. Moreover, the proportion of PD-L1-expressing ICs may depend on the level of infiltration of immune cells into the tumor microenvironment. This may differ between different sections of the tumor, therefore contributing to the observed heterogeneity of IC PD-L1 expression. Variability in IC scoring may also be due to a pathologist’s technical ability in scoring ICs. Studies have noted that IC scoring is more variable than TC scoring when different pathologists assess identical sections [23, 37], suggesting a need for more extensive training of pathologists specifically on scoring of ICs. IC results in NSCLC should not be extrapolated to more immunogenic cancers such as UC, where there are generally higher proportions of patients with high IC PD-L1 expression (e.g. in the study by Massard et al. using the VENTANA SP263 assay, 45% of screened UC patients were found to be PD-L1-positive on the basis of IC expression, using a 25% cut-off [38]).

Our study investigated PD-L1 expression using only the VENTANA PD-L1 (SP263) assay. Similar studies have been carried out using the other approved PD-L1 assays and have been published by the US Food and Drug Administration (FDA) as part of the approval process for each assay (Table 6) [24–27]. PD-L1 expression in TCs has been assessed with the Dako PD-L1 IHC 22C3 PharmDx (intra-block and intra-case concordance: both 100% at the ≥50% cut-off, in NSCLC) [24] and the Dako PD-L1 IHC 28–8 PharmDx assay (intra-case concordance: 94% each at the ≥1%, ≥5% and ≥ 10% cut-offs, in NSCLC) [25]. PD-L1 expression has been assessed using the VENTANA PD-L1 (SP142) assay for ICs in UC (intra-block and intra-case concordance: 100 and 91%, respectively, at the ≥5% cut-off) [27] and for TCs and ICs in NSCLC (intra-block and intra-case concordance: 96 and 81%, respectively, at the ≥50% TC or ≥ 10% IC cut-offs) [26] (Table 6) [24–27]. Our data are broadly consistent with these reports, supporting the notion that a patient’s TC PD-L1 classification is unlikely to be altered under routine clinical sampling protocols. This is further supported by the Rehman et al. study, which showed minimal intra-case heterogeneity in PD-L1 staining of TCs in 35 NSCLC cases, and suggested that staining one block of a tumor should be enough to represent the entire tumor [23].

**Table 6:** Data on intra-block and intra-case concordance in PD-L1 classification, from publicly available FDA documents^a^

Assay	TC PD-L1 staining % *OPA (% cut-off)*	IC PD-L1 staining % *OPA (% cut-off)*	TC or IC PD-L1 staining % *OPA (% cut-off)*	n
*Intra-block concordance in PD-L1 classification*				
Dako 22C3				
NSCLC [24]	100% (≥50%)	–	–	20
Dako 28–8 [25]	–	–	–	
VENTANA SP142				
NSCLC [26]	–	–	96% (≥50% TC or ≥ 10% IC)	24
UC [27]	–	100% (≥5%)	–	8
*Intra-case concordance in PD-L1 classification*				
Dako 22C3				
NSCLC [24]	100% (≥50%)	–	–	20
Dako 28–8				
NSCLC [25]	94% (≥1%; ≥5%; ≥10%)	–	–	16
VENTANA SP142				
NSCLC [26]	–	–	81% (≥50% TC or ≥ 10% IC)	27
UC [27]	–	91% (≥5%)	–	22

^a^Summary of Safety and Effectiveness Data (SSED)

*FDA* Food and Drug Administration, *IC* tumor-infiltrating immune cell, *NSCLC* non-small cell lung cancer, *OPA* overall percentage agreement, *PD-L1* programmed cell death ligand-1, *TC* tumor cell, *UC* urothelial carcinoma

A notable strength of our study lies in the analysis of two different sections from the same tumor that were cut 7 months apart (for the 200 NSCLC cases). This mimics what might occur in the clinical setting, where an additional section may be requested from the same tissue block several months later. The high concordance observed in the analysis of TCs here gives a high level of confidence in the reliability of PD-L1 scoring in the real-life clinical situation.

Moreover, our study investigated the consistency in PD-L1 scoring of both TCs and ICs, and using a wide range of clinically relevant cut-offs. The cut-offs were chosen based on the diagnostic algorithms that have been approved or are currently being investigated for the different PD-L1 diagnostic assays and anti-PD-1 / anti-PD-L1 therapies (Table 1) [4, 8, 10–13, 17, 19–22, 30–32].

One limitation of our study is the fact that the FFPE samples used came from large tumor resections instead of biopsies, thus may not be representative of all clinical samples. This was done for practical reasons, as a large amount of tissue was required (to cut 51 sections per sample), which could not have been achieved from a small biopsy. Whether the PD-L1 status of a tumor would vary depending on the sample type (cytology vs biopsy vs resection) is unknown. A number of studies have investigated concordance in PD-L1 expression between different types of samples using validated FDA approved PD-L1 tests [39–41]. Ilie et al. reported discordance of 19% between TC scoring in resections and biopsies, with notably higher discordance when IC scoring was also taken into account. This study used the VENTANA PD-L1 (SP142) assay, which has shown lower analytical sensitivity than SP263 [42, 43]. Skov et al. and Heymann et al. both found strong concordance between resections and small biopsies and/or cytology samples using the Dako PD-L1 IHC 22C3 PharmDx and/or PD-L1 IHC 28–8 PharmDx assays [40, 41], which have shown similar sensitivity to SP263 [29, 42].

A second limitation of our study was the small sample size of HNSCC and UC cases analyzed (only five cases of each). This may be too small a dataset to confidently draw any conclusions about these indications specifically; however, the results from the NSCLC small intra-block and intra-case study are supported by those from the much larger NSCLC dataset, giving confidence that our findings, particularly those relating to PD-L1 staining of TCs, can be applied across indications.

Thirdly, the scoring of PD-L1 expression in our study was carried out by a single pathologist. This approach was taken to allow determination of intra-block and intra-case agreement without confounding variables. However, in clinical practice samples may be scored by different pathologists, and it would, therefore, be important to establish whether inter-pathologist variability would impact the results.

## Conclusions

Our study showed high intra-block and intra-case concordance in TC PD-L1 classification with the VENTANA PD-L1 (SP263) assay, at various applied cut-offs. These data provide confidence in use of this assay to determine a patient’s TC PD-L1 classification, as the results were consistent across the depth of the tumor block and between resections taken from different areas of the tumor. Although more variable than TC staining, consistent IC PD-L1 classification was also observed within and between tumor blocks for most patients.

These are important data to have in hand as the value of biomarker (PD-L1) testing in immunotherapy becomes clearer, and suggest that PD-L1 classification based on the analysis of a single tumor section can be used confidently to inform treatment decisions.

## Additional file

Additional file 1: PD-L1 scoring of 15 NSCLC, HNSCC or UC cases. (DOCX 22 kb)

## Abbreviations

CI: Confidence interval
CLIA: Clinical Laboratory Improvement Amendments
FDA: Food and Drug Administration
FFPE: Formalin-fixed, paraffin-embedded
H & E: Hematoxylin and eosin
HNSCC: Head and neck squamous cell carcinoma
IC: Tumor-infiltrating immune cells
NPA: Negative percentage agreement
NSCLC: Non-small cell lung cancer
OPA: Overall percentage agreement
PD-1: Programmed cell death-1
PD-L1: Programmed cell death ligand-1
PPA: Positive percentage agreement
TC: Tumor cell
UC: Urothelial carcinoma
