# Supplementary material for: Consistency of tumor and immune cell programmed cell death ligand-1 expression within and between tumor blocks using the VENTANA SP263 assay
Source: Diagn Pathol. 2018 Jul 24;13:47. doi: 10.1186/s13000-018-0725-9 (PMC6058354; doi:10.1186/s13000-018-0725-9)
Supplement: Supplementary file 1 — PD-L1 scoring of 15 NSCLC, HNSCC or UC cases. (DOCX 22 kb) [file 13000_2018_725_MOESM1_ESM.docx]

Additional file 1

PD-L1 scoring of 15 NSCLC, HNSCC or UC cases

| Case ID | Slide ID | Sample type | % of PD-L1 positive TC within the tumor area | % of PD-L1 positive IC within the tumor area |
| --- | --- | --- | --- | --- |
| AVD-6Y-7-0865 | 6Y-7-0865A-2 | Lung | 100% | 1–4% |
|  | 6Y-7-0865A-25 | Lung | 100% | 1–4% |
|  | 6Y-7-0865A-50 | Lung | 100% | 1–4% |
|  | 6Y-7-0865B-2 | Lung | 95% | 1–4% |
|  | 6Y-7-0865B-25 | Lung | 100% | 1–4% |
|  | 6Y-7-0865B-50 | Lung | 95% | 1–4% |
| AVD-8Y-4-0362 | 8Y-4-0362A-2 | Lung | 55% | 5–9% |
|  | 8Y-4-0362A-25 | Lung | 60% | 10% |
|  | 8Y-4-0362A-50 | Lung | 55% | 5–9% |
|  | 8Y-4-0362B-2 | Lung | 40% | 10% |
|  | 8Y-4-0362B-25 | Lung | 40% | 5–9% |
|  | 8Y-4-0362B-50 | Lung | 40% | 5–9% |
| AVD-8Y-5-0200 | 8Y-5-0200A-2 | Lung | 95% | <1% |
|  | 8Y-5-0200A-25 | Lung | 95% | <1% |
|  | 8Y-5-0200A-50 | Lung | 95% | <1% |
|  | 8Y-5-0200B-2 | Lung | 85% | <1% |
|  | 8Y-5-0200B-25 | Lung | 90% | <1% |
|  | 8Y-5-0200B-50 | Lung | 90% | <1% |
| AVD-7Y-3-2858 | 7Y-3-2858A-2 | Lung | 65% | 1–4% |
|  | 7Y-3-2858A-25 | Lung | 70% | 1–4% |
|  | 7Y-3-2858A-50 | Lung | 70% | 1–4% |
|  | 7Y-3-2858B-2 | Lung | 85% | 1–4% |
|  | 7Y-3-2858B-25 | Lung | 85% | 1–4% |
|  | 7Y-3-2858B-50 | Lung | 85% | 1–4% |
| AVD-7Y-3-2884 | 7Y-3-2884A-2 | Lung | <1% | 5–9% |
|  | 7Y-3-2884A-25 | Lung | <1% | 5–9% |
|  | 7Y-3-2884A-50 | Lung | <1% | 10% |
|  | 7Y-3-2884B-2 | Lung | <1% | 5–9% |
|  | 7Y-3-2884B-25 | Lung | <1% | 5–9% |
|  | 7Y-3-2884B-50 | Lung | <1% | 1–4% |
| AVD-1R-6-0070 | 1R-6-0070A-2 | Tonsil | 75% | 5–9% |
|  | 1R-6-0070A-25 | Tonsil | 75% | 5–9% |
|  | 1R-6-0070A-50 | Tonsil | 75% | 10% |
|  | 1R-6-0070B-2 | Tonsil | 95% | 10% |
|  | 1R-6-0070B-25 | Tonsil | 95% | 10% |
|  | 1R-6-0070B-50 | Tonsil | 95% | 15% |
| AVD-1R-7-0051 | 1R-7-0051A-2 | Tongue | 35% | 5–9% |
|  | 1R-7-0051A-25 | Tongue | 35% | 5–9% |
|  | 1R-7-0051A-50 | Tongue | 25% | 5–9% |
|  | 1R-7-0051B-2 | Tongue | 30% | 5–9% |
|  | 1R-7-0051B-25 | Tongue | 30% | 5–9% |
|  | 1R-7-0051B-50 | Tongue | 25% | 5–9% |
| AVD-7R-0-0069 | 7R-0-0069A-2 | Larynx | <1% | 1–4% |
|  | 7R-0-0069A-25 | Larynx | <1% | 1–4% |
|  | 7R-0-0069A-50 | Larynx | <1% | 1–4% |
|  | 7R-0-0069B-2 | Larynx | <1% | <1% |
|  | 7R-0-0069B-25 | Larynx | <1% | <1% |
|  | 7R-0-0069B-50 | Larynx | <1% | <1% |
| AVD-7R-0-0071 | 7R-0-0071A-2 | Tonsil | 40% | 1–4% |
|  | 7R-0-0071A-25 | Tonsil | 40% | 1–4% |
|  | 7R-0-0071A-50 | Tonsil | 40% | 1–4% |
|  | 7R-0-0071B-2 | Tonsil | 40% | 1–4% |
|  | 7R-0-0071B-25 | Tonsil | 40% | 1–4% |
|  | 7R-0-0071B-50 | Tonsil | 40% | 1–4% |
| AVD-7R-3-0004 | 7R-3-0004A-2 | Tongue | 100% | 1–4% |
|  | 7R-3-0004A-25 | Tongue | 100% | 1–4% |
|  | 7R-3-0004A-50 | Tongue | 100% | 1–4% |
|  | 7R-3-0004B-2 | Tongue | 100% | 1–4% |
|  | 7R-3-0004B-25 | Tongue | 100% | 1–4% |
|  | 7R-3-0004B-50 | Tongue | 100% | 1–4% |
| AVD-6Y-9-0479 | 6Y-9-0479A-2 | Bladder | 100% | 5–9% |
|  | 6Y-9-0479A-25 | Bladder | 95% | 5–9% |
|  | 6Y-9-0479A-50 | Bladder | 95% | 5–9% |
|  | 6Y-9-0479B-2 | Bladder | 95% | 5–9% |
|  | 6Y-9-0479B-25 | Bladder | 95% | 5–9% |
|  | 6Y-9-0479B-50 | Bladder | 100% | 5–9% |
| AVD-8Y-2-0046 | 8Y-2-0046A-2 | Bladder | <1% | <1% |
|  | 8Y-2-0046A-25 | Bladder | <1% | <1% |
|  | 8Y-2-0046A-50 | Bladder | <1% | <1% |
|  | 8Y-2-0046B-2 | Bladder | <1% | <1% |
|  | 8Y-2-0046B-25 | Bladder | <1% | <1% |
|  | 8Y-2-0046B-50 | Bladder | <1% | 1–4% |
| AVD-8Y-2-0831 | 8Y-2-0831A-2 | Bladder | 1–4% | <1% |
|  | 8Y-2-0831A-25 | Bladder | 1–4% | <1% |
|  | 8Y-2-0831A-50 | Bladder | 1–4% | <1% |
|  | 8Y-2-0831B-2 | Bladder | 1–4% | <1% |
|  | 8Y-2-0831B-25 | Bladder | 1–4% | <1% |
|  | 8Y-2-0831B-50 | Bladder | 1–4% | 1–4% |
| AVD-8Y-3-0763 | 8Y-3-0763A-2 | Bladder | 100% | 30% |
|  | 8Y-3-0763A-25 | Bladder | 100% | 30% |
|  | 8Y-3-0763A-50 | Bladder | 100% | 30% |
|  | 8Y-3-0763B-2 | Bladder | 100% | 30% |
|  | 8Y-3-0763B-25 | Bladder | 100% | 30% |
|  | 8Y-3-0763B-50 | Bladder | 100% | 30% |
| AVD-8Y-5-0842 | 8Y-5-0842A-2 | Bladder | 70% | 1–4% |
|  | 8Y-5-0842A-25 | Bladder | 70% | 5–9% |
|  | 8Y-5-0842A-50 | Bladder | 75% | 1–4% |
|  | 8Y-5-0842B-2 | Bladder | 75% | 1–4% |
|  | 8Y-5-0842B-25 | Bladder | 75% | 5–9% |
|  | 8Y-5-0842B-50 | Bladder | 75% | 5–9% |

TC and IC PD-L1 expression scores from the three sections of each block from 15 NSCLC, HNSCC or UC cases (two blocks per case; five cases per tumor type)

HNSCC: head and neck squamous cell carcinoma; IC: tumor-infiltrating immune cells; NSCLC: non-small cell lung cancer; PD-L1, programmed cell death ligand-1; TC: tumor cells; UC: urothelial carcinoma
